# Effect of Pertubation on Pregnancy Rates before Intrauterine Insemination Treatment in Patients with Unexplained Infertility

**Published:** 2014-03-09

**Authors:** Funda Yildiz, Nuray Bozkurt, Ahmet Erdem, Mehmet Erdem, Mesut Oktem, Recep Onur Karabacak

**Keywords:** Pertubation, Gonadotrophin, Unexplained Infertility

## Abstract

**Background:**

The aim of this study was to determine the relationship between marital violence and distress level among women with a diagnosis of infertility.

**Materials and Methods:**

In this prospective randomized study, a total of 180 patients were included in the study. Amongst these, pertubation of the uterine cavity was carried out in 79 patients prior to insemination. One patient in the pertubation group was later excluded because insemination could not be performed due to cycle cancellation.

**Results:**

There were no significant differences in demographic characteristics between the study and control groups. When the pregnancy rates of both groups were evaluated, 14(17.8%) patients in the study group achieved pregancy. Three (3.8%) had a biochemical pregnancy, 1(1.3%) miscarried and 10(12.7%) had live births. In the control group, a total of 24(23.8%) pregnancies were achieved, amongst which one (1%) had a biochemical pregnancy, 3(3%) miscarried and 20(19.8%) resulted in live births. There was no significant difference between groups in terms of total pregnancy and live birth rates (p>0.05). There was a 21% total pregnancy loss rate. There was no significant difference between the control and study groups in terms of pregnancy loss rates (p>0.05).

**Conclusion:**

This study on a homogenous group of unexplained infertile patients determined that the addition of pertubation to a controlled ovarian hyperstimulation plus intrauterine insemination (COH+IUI) treatment protocol did not affect pregnancy rates (Registration Number: NCT01999959).

## Introduction

Couples that fail to concieve despite regular intercourse for at least one year are evaluated for infertility. The workup of such patients include basic infertility tests. These tests involve the spermiogram, a marker of sperm production, a hysterosalpingogram (HSG) which determines tubal patency, and the evaluation of ovulation. The pregnancy rate in normally fertile couples is 20-25%, while this rate averages between 2-4% in infertile couples (1). Since controlled ovarian hyperstimulation plus intrauterine insemination (COH+IUI) is less expensive and less laborious than intracytoplasmic sperm injection or *in vitro* fertilization (ICSI/IVF), the former is considered as a first treatment choice. Studies to increase the suc- cess of this treatment modality are ongoing.

Hysterosalpingography, which is one of the basic tests of infertility, has a mechanical washing effect on the uterine cavity and tubes. Particularly after performing this test with an oil-based contrast medium, the chance of spontaneous pregnancy increases. Given that the HSG is normal, expectant management reveals a pregancy rate of up to 40% in such patients. If the same patients were to undergo appropriate treatment, the pregnancy rate generally reaches 30%. Therefore, following an HSG with an oil-based contrast, management with an average duration of six months is preferred in most centers (2)

The positive effect of this phenomenon may be utilized by introducing uterine cavity pertubation prior to infertility treatment. Considering cases of early stage endometriosis, the mechanical effect of pertubation may decrease minor tubal adhesions. The proposed immunologic effect is based on the prevention of sperm phagocytosis and the removal of peritoneal cytokins and immunological factors (3)

We considered the mechanical and immunological effect of uterine cavity pertubation on treatment protocols for patients with unexplained infertility. To demonstrate a possible beneficial impact of this procedure we designed a randomized prospective study, in which uterine washing was administered prior to insemination in patients diagnosed with unexplained infertility.

## Materials and Methods

This study was carried out in patients who presented to Gazi University Hospital, Division of Infertility Services with diagnoses of unexplained infertility. This was a single-center, prospective, randomized, blinded control trial undertaken at a tertiary care university fertility center between January 2010-March 2011

Patients who fulfilled the inclusion criteria (180 cases) were randomized by systemic randomization in which they were sequentially allocated to two treatment groups. This systemic randomization was performed by the nurse coordinator on the hCG injection day in the absence of the clinicians.

We included 180 patients in the study. It was initially planned to form to equal groups, namely the control and study groups, however only 79 eligible participants gave writen consent and accepted the pertubation procedure. The other 101 participants received the planned treatment protocol only (control group). We excluded one patient from the study group due to cycle cancellation. The study group eligibility criteria included: age of 18-44 years; presence of regular menstrual cycles and ovulation; absence of tubal occlusion on HSG; sperm concentration >15 million spermatoza/ml and total sperm number >39 million/ ml according to WHO criteria (4). Exclusion criteria were the presence of endocrinologic disease; use of nonsteroidal anti-inflammatory drugs (NSAIDs) or corticosteroids; clinical findings suggestive of pelvic inflamatory disease; and the presence of undiagnosed uterine bleeding.

Follitropin alpha (Gonal F, rec-FSH, Serona, Turkey), follitropin beta (Puregon, rec-FSH Organon, Turkey), urinary hMG (Merional, Aris, Turkey veya Menogon, Erkim, Turkey) and urofolitropin (Fostimon, Aris, Turkey) were used for ovarian stimulation. Ovulation induction was started between 2-5 days of menstruation on patients who had no residual cysts larger than 15 mm as visualized with basal transvaginal USG (ultrasound). All patients had 75-150 Iu/day drug as an initial dose. On cycle day 5-6, stimulated follicles were measured ultrasonographically. Induction doses were increased or decreased between 37.5-75 IU/day according to follicle size. Blood estriol and LH levels were monitored and recorded during follow up. When 1-2 follicles reached a mean diameter of 17 mm, we administered 250 µg of recombinant hCG to trigger ovulation. Treatment was discontinued when two or more follicles showed equal maturation in order to avoid the risk of multiple pregnancies. In case of maturation of two follicles because of the risk of a twin pregnancy, treatment was discontinued. At 35-36 hours after the hCG injection, IUI was performed. Two days following insemination micronized vaginal progesterone (400-600 mg/day, Progestan, Kocak, Turkey) was administered to support the luteal phase until the pregnancy was confirmed. Two weeks after insemination, blood beta-hCG levels were analyzed. If the result was negative, progesterone support was discontinued. Patients with positive hCG titers received progesterone support until nine weeks of gestation.

The swim up prcedure was used for sperm washing. A Rocket Embryo IUI catheter was used during insemination.

Since our study aimed to evaluate the use of pertubation prior to ovulation, the procedure was performed on the same day as the hCG injection, prior to its administration. The pertubation procedure was performed in the dorsal lithotomy position after the application of a vaginal speculum. The vaginal portion of the cervix was cleansed with a povidone-iodine (PVP-I) solution to prevent potential uterine infections. Povidone-iodine is a stable chemical complex of polyvinylpyrrolidone (povidone, PVP) and elemental iodine (I). It is used for the prevention and treatment of skin infections, and the treatment of wounds. This solution has cytotoxic effects on sperm and embryo. However PVP-I is used on the hCG injection day, 36 hours before IUI. Similarly, many clinics use betadine for cervical preparation prior to the oocyte pick-up procedure. Vaginal preparation by betadine does not seem to affect the IVF results (5).

Uterine washing was accomplished by introducing a silicone catheter through the internal cervical os, after which 20 cc saline and 1 cc jetocain were slowly injected. Special attention was given to infuse the solution over a few seconds, since rapid injections could give rise to pelvic pain. Jetocain was used for its local anesthetic effects. The speculum was removed and the procedure completed after the injection.

Two weeks after insemination, a blood beta hCG level was obtained. Two days later, patients whose results were positive had a repeat test to ascertain a healthy increase in beta hCG levels. Patients whose control beta hCG level decreased or those who experienced vaginal bleeding were classified as biochemical pregnancies. Patients with a healthy beta hCG increase were evaluated two weeks later for clinical pregnancy status. A regular intrauterine gestational sac and presence of fetal cardiac activity confirmed the clinical pregnancy. Patients who experienced pregnancy loss after the sac was visualized were considered as clinical miscarriages. Pregnancies over 20 gestational weeks that resulted in births were defined as live births.

This study was approved by the Ethics Committee clinical studies in Ankara and received approval on 25.11.2009.

Pertubation was considered the independent variable. Pregnancy rate (chemical and clinical) was the primary dependent outcome variable.

Data were analyzed with the SPSS software version 15.0 for Windows (SPSS Inc., Chicago, Illinois, USA). Continuous variables (age, duration of infertility, total motile sperm count, 3^rd^ day FSH, initial and total dose of ovulation induction agent, follicle count and size, and endometrial thickness) were presented as mean ± SD. Categorical variables (alcohol or cigarette use, agent of ovulation induction, fertilization rate or pregnancy outcomes) as frequency and percentage. Student’s t test was used to compare normally distributed continuous variables and the Mann-Whitney U test for variables without normal distribution. Categorical variables were compared using the chi-square test. A two-tailed p value of <0.05 was considered statistically significant.

## Results

Among the 180 patients included, 135 were primarily infertile. There were 51 (64%) primary infertile patients in the study group and 84 (83%) primary infertile patients included in the control group. In a comparison between groups, we noted that secondary infertility was more common in the control group (p<0.05).

Participants included in the study were be- tween the ages of 18-44 years. The mean age of the study group was 28.8 ± 5.3 years and 28.2 ± 4.7 for the control group (p=0.401). The average infertility period was 3.6 ± 2.3 years in the study group and 3.8 ± 2.8 years in the con- trol group. This difference was not statistically significant (p=0.684). As seen in table 1, day-3 basal FSH levels were not significantly different between groups (p>0.05).

There were 21 smokers in the study, of which 7 (8.9%) were from study group and 14 (13.9%) were in the control group. There was no statistical difference between both groups when compared for distribution of smokers (p=0.300). Both groups did not include patients that had a background of regular alcohol use.

There was no significant difference between the types and initial or total doses of gonadotrophins (p>0.05, Tables 2, 3). There was also no significant difference between the study and control groups in terms of dominat follicle count, mean follicle diameter and endometrial thickness.

The mean total motile sperm number in the study group was 99.706 ± 85.214 and for the control group, it was 86.304 ± 61.057. A comparison of both groups showed no significant differnce in total motile sperm count (p=0.405).

**Fig 1:**
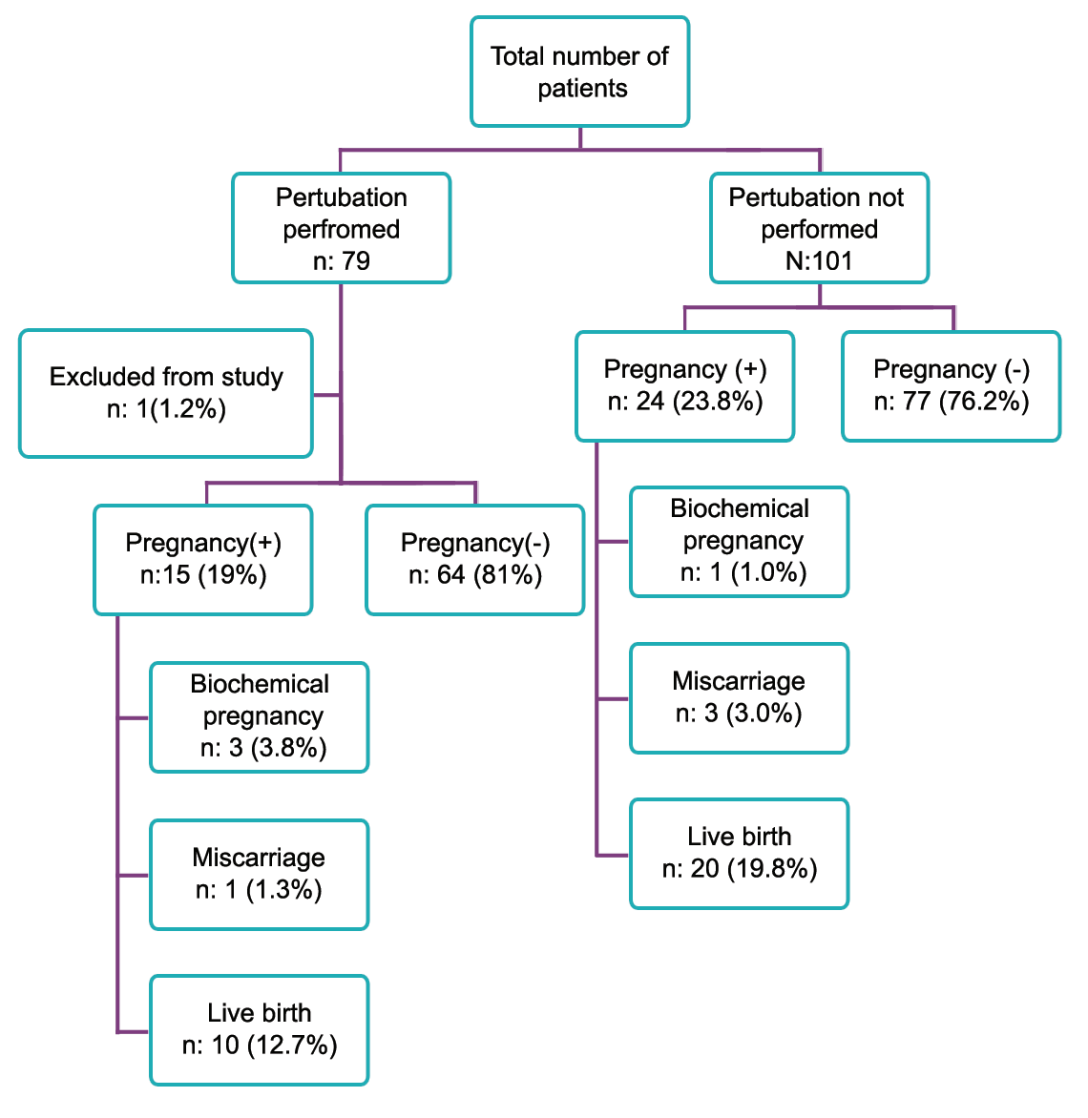
Distribution of pregnancy outcomes.

From the 180 participants, 39 concieved. A total of 15 pregnancies were from the study group and 24 from the control group. In the study group 3 patients had biochemical pregnancies, 1 miscarried and 10 patients had live births. In the control group, 1 patient had a biochemical pregnancy, 3 patients miscarried and 20 patients had live births. Between the two groups, there was no significant difference in pregnancy rates (p=0.296, Tables 4, Fig 1). Pregnancy loss rates were statistically similar.

**Table 1:** Patients’ demographic characteristics


Demographic data	Pertubation		P value	Minimum/maximum
	Performed Mean ± SD	Not performed Mean ± SD		

**Female age (Y)**	28.8 ± 5.3	28.2 ± 4.7	0.401	18-44
**Duration of infertility (Y)**	3.6 ± 2.3	3.8 ± 2.8	0.684	1-17
**3^r^^d^ day FSH(mU/mL)**	5.9 ± 1.7	5.8 ± 1.8	0.827	0.8-15.1


**Table 2:** Distribution of gonadotropins within groups


Pertubation	Agent used in ovarian inductionn (%)			
	rec–FSH	Urinary hMG	rec–FSH+ urinary hMG	Urofollitropin

**Pertubation performed (study group)**	66 (83.5)	5 (6.3)	2 (2.5)	6 (7.6)
**Pertubation not performed (control group)**	73 (72.3)	11 (10.9)	1 (1.0)	16 (15.8)
**Total**	139 (77.2)	16 (8.8)	3 (1.6)	22 (12.2)


**Table 3:** İnitial and total dose of ovulation induction agents


Agents	Pertubation		p-value	Minimum/maximum
	Performed Mean ± SD	Not performedMean ± SD		

**İnitial dose(IU/day)**	84.5 ± 32.7	83.2 ± 27.4	0.962	75-300
**Total dose(IU)**	877.5 ± 469	784.7 ± 31.5	0.385	375-2997.5


**Table 4:** Pregnancy results


Pregnancy	Pertubation		P value
	Performed n (%)	Not performed n (%)	

**None**	64 (81.0)	77 (76.2)	0.440
**Biochemical**	3 (3.8)	1 (1.0)	0.205
**Abortion**	1 (1.3)	3 (3.0)	0.441
**Live births**	10 (12.7)	20 (19.8)	0.202
**IUI not performed**	1 (1.2)	0 (0)	0.257


## Discussion

Infertility affects 10-15% of the reproductive age group (6). Around 10% of the infertile population is classed as unexplained. Ovulation induction and intrauterine insemination is the accepted first line treatment plan for unexplained infertility.

In order to obtain a homogenous patient population we only included patients diagnosed with unexplained infertility in this study.

At least three cytokines are synthesized by the endometrium, colony stimulating factor-1 (CSF-1), leukemia-inhibitory factor (LIF) and interleukin-1 (IL-1), which are associated with implantation (7). CSF-1 expression from endometrium and preimplantation embryo. Cadherin is an important agent for intercellular junctional providing on epithelial cells. In the peri-implantation phase, E-cadherin and E-cadherin mRNA expression from endometrium (8). E-cadherin and E-cadherin mRNA levels are lower in the proliferative endometrium than during the secretion phase. The adhesive function of the endometrium is to be activated after ovulation.

T helper (Th) 1 and 2 expression increases in peripheral lymphocytes of patients with recurrent artificial reproductive tecnology (ART) failure (9). In pregnancy, Th 2 concentration is higher than Th 1 concentration. The Th1/Th2 rate is higher in patients who have recurrent abortions and recurrent implantation failure when compared with a fertile control group (10). In some studies, findings have shown increases in the numbers of peripheral natural killer cells. However this finding has not been fully verified (11).

We can analyze the effect of immunological factors on implantation success in patients with hydrosalpinx. The liquid of the hydrosalpinx blocks implantation either by a direct embrotoxic effect, a negative impact on the endometrium, and mechanical impact. Implantation and pregnancy rate is lower in patients with hydrosalpinx than in a normal control group (12). Prospective rondomized studies have shown that the success of an ART procedure increases with salpingectomy in patients who have hydrosal- pinx. This effect is the same on both of the first ART cycles and with recurrent ART cycles (13, 14). The endometrial environment becomes more ideal for implantation with cleaning of embry- otoxic cytokines. In a similar way, during the pertubation, thin adhesions in the endometrial cavity was opened with rapid fluid pressure.

The downfall of the current study was the use of an open randomized technique during patient recruitment. As a result when we compared both groups, it was evident that in the study group secondary infertile patients outnumbered primary infertile couples, whereas in the control group primarily infertile patients were more common. According to one study performed at our center, independent factors which increased clinic pregnancy rates were secondary infertiliy and unexplained infertility. However these factors did not affect live birth rates (15).

Although the pregnancy rate was higher in the control group, this was not statistically significant when compared with the study group (p=0.296). When we evaluated both biochemical and clinical pregnancies, the pregnancy rate was 17.8% in the study group and 23.8% in the control group. The rate of live births, which was the main purpose of this treatment was 12.7% in the study group and 19.8% in the control group.

Spontaneous miscarriage occurs in 15-20% of known pregnancies. If serial hCG is measured to detect early subclinical pregnancy loss, this rate would increase to 30% (16, 17). In our study, 21% of total pregnancies were abort. This rate was not higher than the expected pregnancy loss rates in normal cycles. When we compared pregnancy loss rates in both groups, there was no significant difference observed (p>0.05).

Aboulghar et al. included 213 patients in a study where they performed hydropertubation on 103 patients. They used clomiphen citrate and urinary HMG for ovulation induction followed by IUI. In our study, we only used gonadotropins for ovulation induction. Both studies have performed intrauterine insemination after ovulation induction. Generally the expected fecundability rate associated with this type of treatment protocol is approximately 17% (18). Aboulghar et al. reported an ongoing pregnancy rate of 12.6% in their study group. Similarly, our research resulted in a rate of fecundability of 17.8% and an ongoing pregnancy rate of 12.7% in the study group. Therefore our results were compatible with the aforemenioned study (19).

In our study the control group’s fecundability rate was 23.8% and the continued pregnancy rate was 19.8%. There was no significant difference between the study and control groups. However the relatively higher rate in the control group suggested the negative effects of pertubation.

Yapça et al. investigated the effectivity of hydrotubation in unexplained infertility therapy by evaluating 80 patients and 144 cycles (20). They reported 11(15.7%) pregnancies and 9(12.86%) clinical pregnancies in a total of 70 cycles in the study group. A total of 74 cycles in the control group yielded 4(5.4%) pregnan- cies and 2(5%) clinical pregnancies. There was a significant overall difference between the two groups (p=0.0219). When evaluated on the basis of individual patients, 11(27.5%?) pregnancies and 9(22.5%) clinical pregnancies occurred following two cycles of therapy in 40 patients of the study group. In the control group of 40 patients, there were 4(10%) pregnancies and 2 (5%) clinical pregnancies following two cycles of therapy. When compared on the basis of individual patients, a significant difference was noted (p=0.0231). As a result, the uterine lavage had a positive effect on treatment success in unexplained infertile subjects. Although this study was similar to ours, we were unable to establish a positive effect of pertubation on fecundability. In contrast to a study by Yapça, the current study included a larger population (180 vs. 80), where each patient underwent a single cycle of treatment.

Lei et al. have reported the effects hydrotubation in 50 formerly proven tubal occlusive patients (21). The hysteroscopic procedure involved the passage of a thin plastic canula through the fallopian tube simulatenously using irrigation media that contained hydrocortisone, gentamycine and procain. The use of additional therapeutic agents in hydrotubation might explain their increased rate of fecundability.

Edelstam et al. performed out a prospective randomized study to evaluate the effect of pertubation on pregnancy rates in patients with unexplained infertility (3). Pertubation was performed prior to ovulation. A total of 130 cycles were investigated. There was a significant difference between the pregnancy rates (14.9 vs. 3.2%) of both groups. The authors concluded that pertubation could be used in conjuction with ovulation induction and intrauterine insemination as a first line management protocol in couples with unexplained infertility.

## Conclusion

In sum, results of this study revealed that pertubation prior to insemination did not effect pregnancy rates.
